# Micropropagation of *Cannabis sativa*: genetic and epigenetic stability assessment over multiple generations

**DOI:** 10.1186/s42238-026-00406-y

**Published:** 2026-02-19

**Authors:** Vincent Lefebvre, Davoud Torkamaneh, Sylvie Deslauriers, Théo Devèze, Maxime de Ronne, Mélodie B. Plourde, Mebarek Lamara, Hugo Germain

**Affiliations:** 1 Groupe de Recherche en Biologie Végétale, Department of Biochemistry, Chemistry, Physics and Forensic Science, Université du Québec À Trois-Rivières (UQTR), 3351 Bd des Forges, Trois-Rivières, QC G8Z 4M3 Canada; 2 Institut de Biologie Intégrative Et Des Systèmes (IBIS), Department of Phytology, Université Laval, Québec, QC G1V 0A6 Canada; 3 Phytoclone Inc, Saint-Étienne-Des-Grès, QC G0X 2P0 Canada; 4 Institut de Recherche Sur Les Forêts (IRF), Université du Québec en Abitibi-Témiscamingue (UQAT), Rouyn-Noranda, QC J9X 5E4 Canada

**Keywords:** *Cannabis sativa*, Micropropagation, Genetic variations, SNPs, Epigenetic variations, Differentially methylated positions, *3D-GBS*, *EM-seq*

## Abstract

**Background:**

Micropropagation is an increasingly sought-after propagation method in the cannabis industry as growers seek to maximize production efficiency, improve multiplication rates and cultivate plants that are free from biotic stress. However, knowledge about the impact of micropropagation on cannabis is still limited in the scientific literature, as most studies have primarily focused on optimizing environmental parameters and growth conditions.

**Methods:**

*In vitro* cultures of three cannabis cultivars (Critical Purple Kush, Green Crack, and Gelato) were initiated and maintained for 60 weeks with subcultures every three weeks. Leaf samples were collected at the start and after every five subcultures for DNA extraction, and the genomes were sequenced using 3D-GBS and EM-seq to identify genetic (SNPs) and epigenetic (DMPs) variations, followed by GO and KEGG pathway analyses.

**Results:**

The results revealed that genomic variants from our CPK (16,169), GC (15,472) and GEL (16,605) samples were mostly located within the intergenic regions. Mutations seem to mostly occur during culture initiation and the first five subcultures, plateauing afterwards up to the 20th subculture. Methylation sequencing revealed that DMPs were less frequent than SNPs, with differential methylation levels showing cultivar-specific patterns varying between CPK, GC and GEL with respectively 22%, 9% and 13% for promoter regions, 13%, 5% and 6% for exon regions, 4%, 4% and 3% for intron regions and 61%, 82% and 78% for intergenic regions. These results indicate a strong cultivar dependency of epimutation and suggest their potential phenotypic impact. GO and KEGG pathway enrichment analyses of genes harboring SNPs and DMPs revealed functional associations relevant to cannabis micropropagation.

**Conclusion:**

Our results highlight the importance of monitoring both genetic and epigenetic stability for long-term cannabis cultivation.

**Supplementary Information:**

The online version contains supplementary material available at 10.1186/s42238-026-00406-y.

## Introduction

Since the legalization of cannabis in several jurisdictions, and more specifically in Canada in October 2018, a multitude of companies started the production of recreational or medical cannabis. Consumers have high standards when it comes to purchasing cannabis, and products with consistent tetrahydrocannabinol (THC) levels of 30% or more are the most sought-after in dispensaries. This implies that producers aiming to thrive in the cannabis market are highly dependent on the reproducibility of their products from one harvest to the next, highlighting the importance of preserving the best phenotypes and avoiding at all costs the various diseases afflicting the industry. However, conventional cultivation methods often overlook critical aspects of sustainable cannabis production, such as the initial innocuity of cultivars, the efficient use of production space, and the long-term preservation of genotypes and phenotypes. Indeed, many companies find themselves facing problems such as pathogen contamination and phenotypic inconsistencies, which can surface after several months or even years of production. However, adopting different approaches could help prevent these issues.

An impressive diversity of cultivation methods can be observed within growth operations, either using soil, coco choir, rock wool, lava rock, hydroponics or aeroponics. However, the same issues affect these systems, mainly mold proliferation in the canopy, insidious virus growth, presence of insects at the roots, loss of vigor and loss of cannabinoid yield (Punja et al. 2024). Due to the high cost of diagnostic tools and the experimental nature of treatment methods, many producers experience losses without knowing the source of their issues or how to address them effectively. To make things right, they must review their production model from top to bottom and put the necessary measures in place to eliminate and prevent these issues. This includes obtaining clean cultivars, routinely checking for cultivar innocuity, maintaining an aseptic environment, preventing cross-contamination between different cultivars and preserving genotypes and phenotypes of interest.

There are a variety of methods that allow the propagation of cannabis on an industrial scale, including the use of seeds for field cultivation and cuttings, the latter being undoubtedly the most popular method among indoor growers. Compared to seeds, the cutting method is more expensive but allows for a more uniform crop with a consistent phenotype, hence producing high-quality flowers that meet consumer requirements and government standards (Lata et al. 2010). However, growers have reported loss of vigor and reduced cannabinoid content after long periods of cutting propagation, due in part to the accumulation of somatic mutations over many generations (Adamek et al. 2022, 2024). Additionally, the maintenance of mother plants in the vegetative stage represents a loss of production space of about 10 to 15%. Moreover, these plant stocks are susceptible to damage caused by insects as well as contamination by pathogens or viruses, which poses a risk to the entire production chain (Caplan et al. 2018; Monthony et al. 2021).

Micropropagation, which consists in mass propagation of plantlets in a controlled aseptic environment, typically in growth vessels on stacked shelves equipped with lighting systems (Shukla et al. 2017), is becoming increasingly coveted as an alternative in the industry. This approach requires less space, which is especially attractive for growers wanting to preserve a wide range of cultivars, whereas the sterile nature of the process facilitates the production of contamination-free plantlets, which is undoubtedly the most prized advantage of this method. Moreover, micropropagation is often considered the first step towards the various biotechnologies that surround cannabis research (Hesami et al. 2021). The ability to regenerate, multiply, and acclimate plantlets from a sterile environment enables researchers to use biotechnological breeding tools and techniques such as agroinfiltration (Deguchi et al. 2020), CRISPR-CAS9 system (Zhang et al. 2021), polyploidy induction (Bagheri et al. 2015; Parsons et al. 2019), somatic embryogenesis (Hesami et al. 2021), and haploid production (Niazian et al*.* 2020). However, in vitro*-*cultured plants are not immune to the development of somaclonal variations, which refer to genetic or epigenetic changes. Such variations, generally considered undesirable from a commercial standpoint, have been reported across all in vitro propagation systems and stages (Kitimu et al. 2015; Adu-Gyamfi et al. 2016; Duta-Cornescu et al. 2023; Johnson et al. 2025).

Somaclonal variations during micropropagation result from the frequent cell division and the exposure of plant tissues to diverse abiotic stresses, such as changes in nutrient composition, plant growth regulators and light intensity (Akomeah et al. 2019; Monthony et al. 2021). This raises the question of how in vitro culture technology compares to propagation by cuttings in terms of preserving the phenotypes of interest.

To address concerns regarding the genetic stability of cannabis during micropropagation, we initiated in vitro cultures of three cannabis cultivars derived from mother plants. These cultures were kept in the vegetative phase on the Can TC multiplicative culture medium with regular transfers to the MS IBA hardening medium. To determine the genetic integrity of these cultivars, we investigated somaclonal variations in each cultivar over an extended period of 60 weeks, with subcultures performed every 3 weeks. Leaves were collected every 5th transplant for DNA extraction and sequencing. There are many techniques used widely to assess genetic variations in cannabis. Most notably, SSR markers are popular because of their low cost and targeted approach, which can be useful in breeding programs (Borin et al. 2025; Cull and Joly 2025). On the other hand, whole genome sequencing (WGS) has been utilized to generate reference genome assemblies such as the CS10 V2.0, the most recent of which being the Pink Pepper genome (Ryu et al. 2024). WGS has also allowed the creation of the first cannabis pangenome (Lynch et al. 2025). However, these methods are expensive and time consuming, hence why optimized versions have been developed for routine applications. Notably, genotyping-by-sequencing (GBS) provides greater coverage than SSR markers while still being cost and time effective (Aina et al. 2025). As for epigenetics, enzymatic methyl sequencing (EM-seq) provides a wide methylation pattern coverage while preserving DNA sequences better than whole genome bisulfite sequencing (WGBS) (Nuttall et al. 2025). We used 3D-GBS technology as it can provide insight of a completely different order of magnitude compared to previous studies using multilocus genetic markers. In addition, we subjected our DNA samples to EM-seq for methylome analysis. Our goal was to identify high-accuracy methylation patterns using the EM-seq method and to establish a relationship between the epigenetic modifications and somaclonal variations arising from plant tissue culture-induced changes over time. By combining EM-seq and 3D-GBS, we aim to obtain an overview of the genetic and epigenetic modifications of cannabis during extended in vitro micropropagation.

## Results

### Nucleotide polymorphisms detected during long-term micropropagation

In this study, we subjected *Cannabis sativa* L. plants to in vitro shoot multiplication (SM) for a 60-week period with transplanting every 3 weeks. Leaf samples were first collected and frozen from the Critical Purple Kush (CPK), Green Crack (GC), and Gelato (GEL) mother plants right before culture initiation, and subsequently from the in vitro plantlets at every 5th transplantation for downstream genomic DNA extraction. The extracted DNA samples were then used for library preparation and sequencing using both triple-digest genotyping-by-sequencing (3D-GBS) and enzymatic-methyl sequencing (EM-seq) methods.

**Fig. 1:**
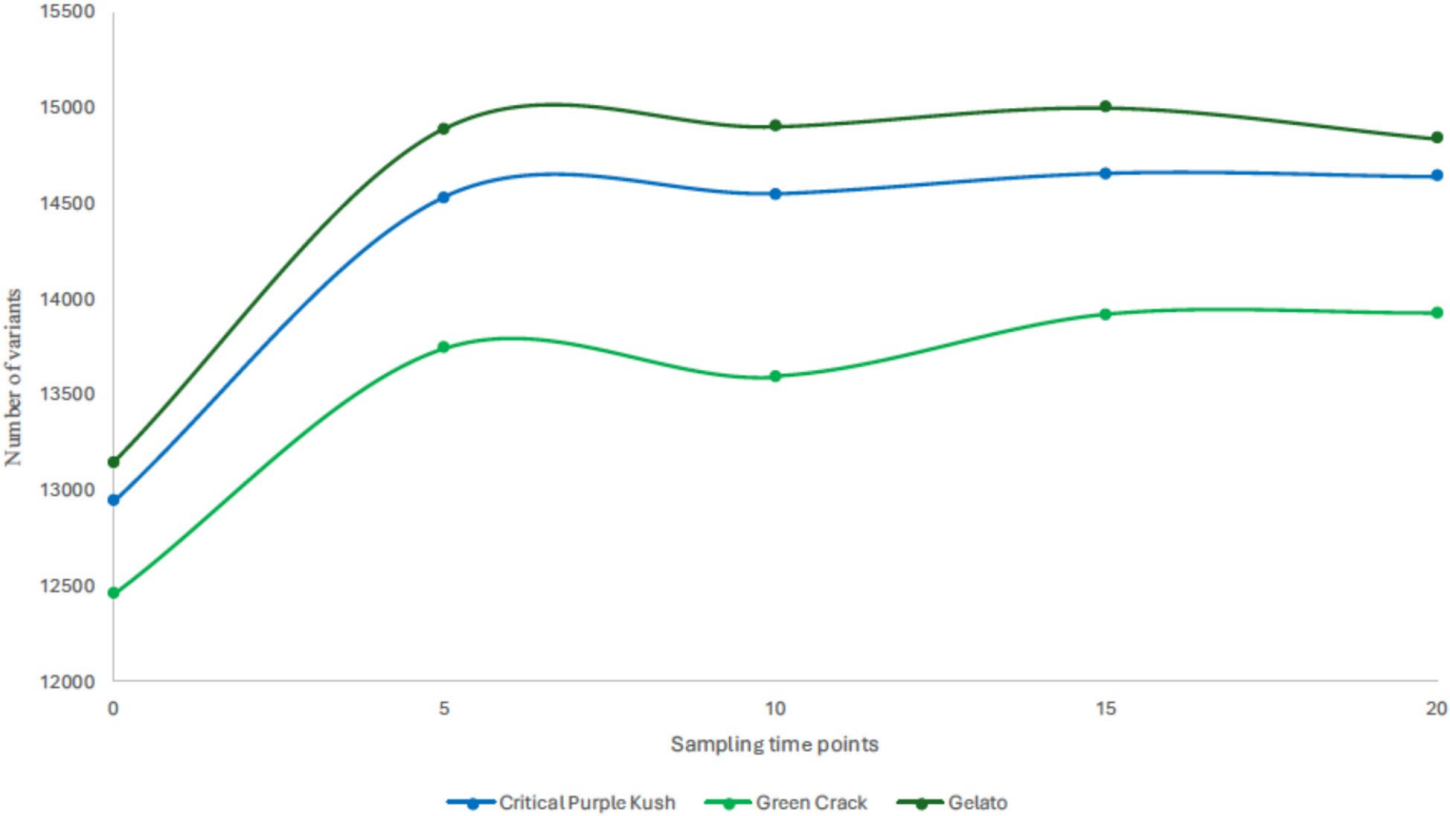
Temporal progression of single nucleotide polymorphisms (SNPs) accumulation detected by 3D-GBS during 60 weeks of in vitro culture in three commercial cultivars

We identified single nucleotide polymorphism (SNP) sites across our three cultivars, with 16,169 found in CPK, 15,472 in GC and 16,605 in Gel (Suppl. Table S1). Figure 1 illustrates the number of SNPs at each sampling point throughout the study for each cultivar. At T = 0, the mother plants harbored 12,943 (CPK), 12,460 (GC) and 13,145 (GEL) SNPs relative to the CS10 v2.0 reference genome (GenBank Accession No. GCA_900626175). The most substantial increase in SNP number occurred between the initiation stage and the 5th transplant, with 1587 new SNPs for CPK (total 14,530), 1284 for GC (total 13,744) and 1743 for GEL (total 14,888).The slight decreases in mutation levels observed at certain time points could be attributed to the fact that we sampled plantlets in batches from multiple growth vessels and that samples from later time points are not necessarily direct descendants from the previous time points but were initiated in parallel; as a result, some clones may have accumulated fewer mutations than others. Even so, the trend indicates that most mutations occur in the early stages of in vitro growth and then plateau or exhibit a very slight steady increase.

### Characterization of genetic variants: distribution, type, location and impact

**Fig. 2:**
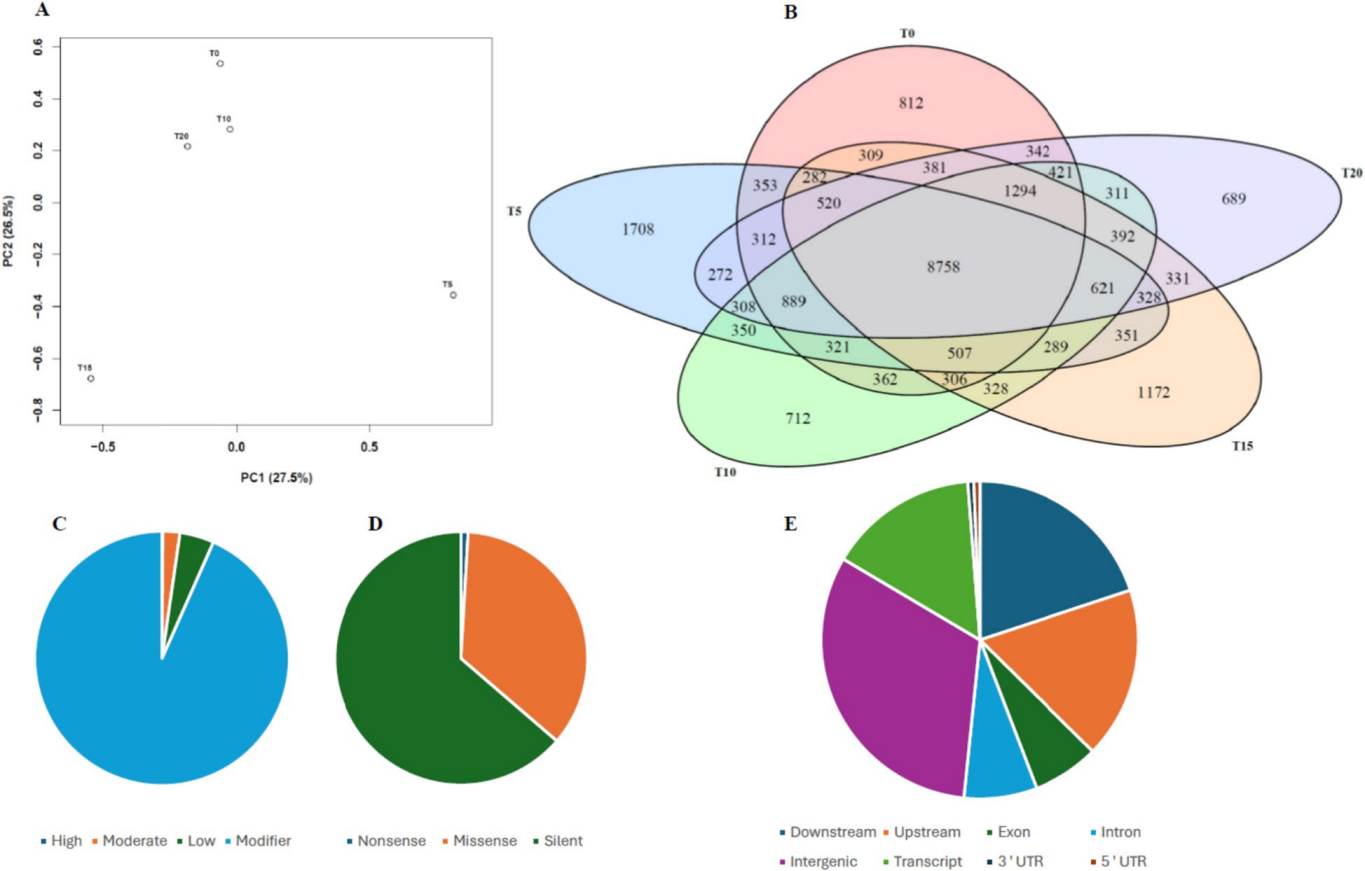
Distribution, impact, types and genomic localization of the genetic variants in the *Cannabis sativa* ‘Critical Purple Kush’ (CPK) cultivar. **A** Principal component analysis showing the clustering of samples across five time points; **B** Venn diagram representing the numbers of shared and unique SNPs between the five time points; **C**-**E** Pie charts showing the distribution of detected SNPs by impact (**C**); **D** type; and genomic localization (**E**) of SNPs across functional regions

In order to better understand the mutational patterns obtained from the 3D-GBS, we performed a Principal Component Analysis (PCA) on our data. For CPK samples, the first two components explained 54% of the total variation, where PC1 is responsible for 27.5%, and PC2 is responsible for 26.5% (Fig. 2A). The PCA reveals that samples from T = 0, T = 10 and T = 20 cluster closely together, whereas T = 5 is more distant along both PC axis, potentially reflecting the mutation spike observed at T = 5 compared to the other time points (Fig. 2A, Suppl. Fig. S1). For GC samples, PC1 and PC2 explained 53.3% of the variation (PC1 = 28.3%, PC2 = 25%). T0 and T15 are grouped, while T5 and T10 show clear separation, indicating time-specific mutational changes (Suppl. Fig. S1). For Gel samples, the first two components accounted for 54.4% of the variation (PC1 = 28.2%, PC2 = 26.2%). T0 and T20 appear closer, whereas T5 and T15 cluster separately, and T10 is more distant, pointing to a unique mutational pattern at this stage (Suppl. Fig. S1).

The number of unique and shared SNPs for the different time points is displayed in a Venn diagram. The number of shared SNPs between all-time points was 8,757 (54.15%). Moreover, T = 5 has the highest number (1,708, 10.56%) of unique mutations, while T = 0, T = 10, T = 15 and T = 20 have 812 (5.02%), 712 (4.40%), 1,172 (7.24%) and 689 (4.26%), respectively (Fig. 2B), thereby reinforcing the idea that the first 5 transplantations induced the most unique mutational changes. Similar patterns were also observed for GC and GEL (Suppl. Fig. S2), with shared SNPs representing 45.54% (GC) and 54.79% (GEL). T5 also had the highest number of unique SNPs in both GC (20.23%) and GEL (6.96%), although the difference was much smaller for Gel.

The impact of a mutation can be estimated based on its type and the genomic regions in which it occurs. Impact analysis of the identified mutations in CPK samples revealed that, as expected, high-impact mutations were the least common (0.1%), followed by moderate impact (2.2%), low-impact (4.4%) and modifiers (93.3%) (Fig. 2C). The same trend was observed for the two other cultivars tested (Suppl. Fig. S3), indicating an overall low mutational impact on the genetic makeup of these plants. Moreover, a substantial 63.7% were silent modifications, whereas 35.4% were labelled as missense and only 0.9% of CPK mutations were deemed to be nonsense (Fig. 2D). Genomic localization of these modifications revealed that intergenic regions were the most affected (31.7%), followed by downstream (19.8%), upstream (17.4%), transcript (15.2%), intron (7.4%), exon (6.8%), 5’ UTR (0.6%) and 3’ UTR (0.6%) regions in CPK (Fig. 2E), meaning that less than 10% of the mutations identified could have had a direct impact on protein-coding sequences.

### DNA methylation changes during micropropagation

**Fig. 3:**
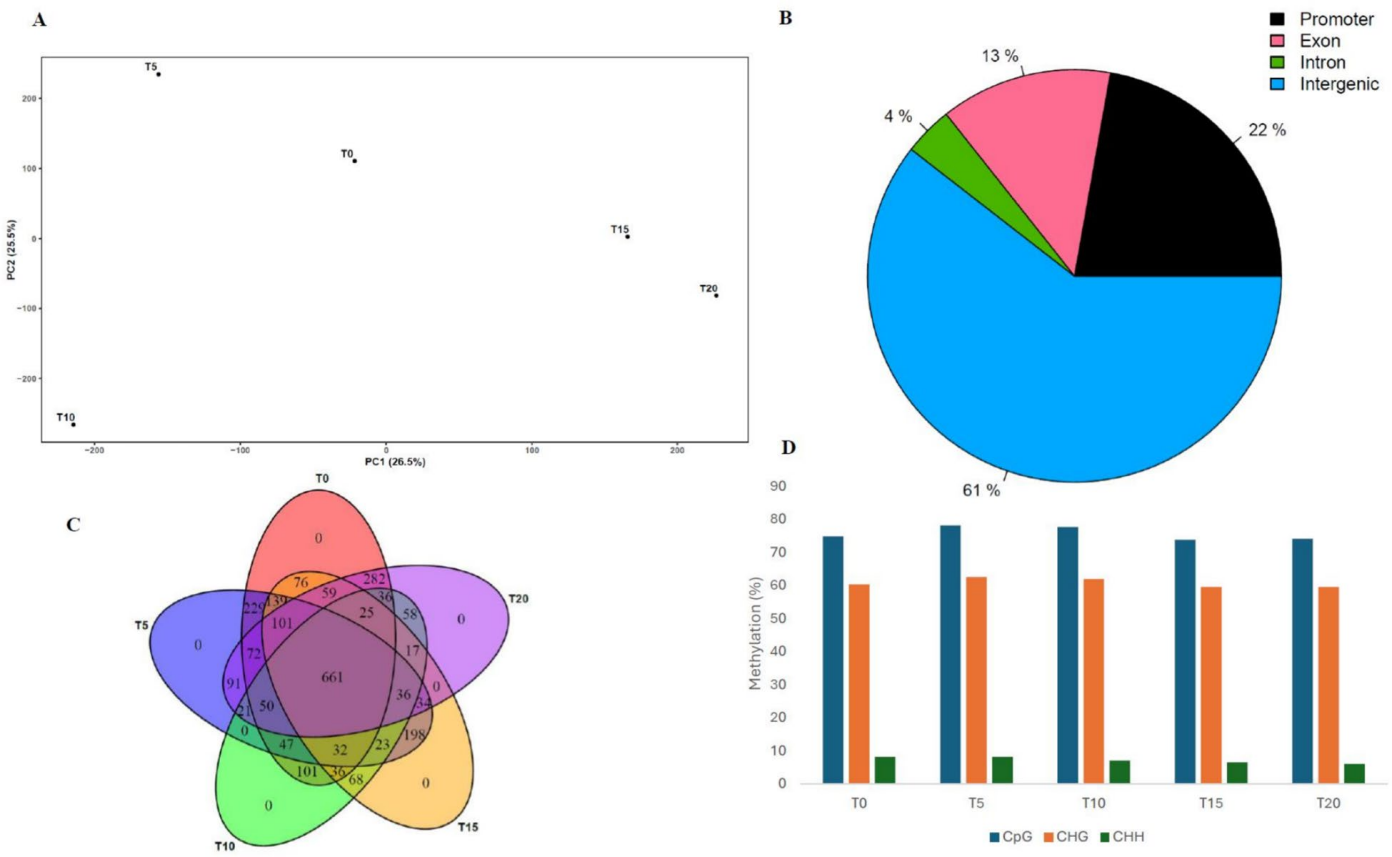
Differential methylation analysis of Critical Purple Kush (CPK) during micropropagation. **A** Principal component analysis of DNA methylation data across subculture cycles; **B** Distribution of differentially methylated positions (DMPs) across the different genomic regions; **C** Venn diagram illustrating comparison of differentially methylated CpGs between the five time points; **D** DNA methylation patterns throughout the subculture cycles

The methylation level analysis of the CpGs sites revealed helpful insight regarding the changes happening to the in vitro grown cannabis’s genetic landscape. For CPK, the PCA (52% of the total variance explained, with PC1 accounting for 26.5% and PC2 for 25.5%) shows a distribution among CPK samples that differs from the pattern observed for somatic mutations, indicating a more consistent progression throughout the study (Fig. 3A). However, this distribution pattern varies vastly between cultivars (Suppl. Fig. S4). Overall, we identified 1,540 CpGs hypermethylation sites in CPK samples. They were more prevalent in intergenic regions (61%), followed by promoter (22%), exon (13%) and intron (4%) regions (Fig. 3B). Differentially methylated positions (DMPs) appear to differ greatly between cultivars, both in numbers (GC 5,974 and GEL 3,471) and localization, with 61% and 78% in intergenic regions, 22% and 13% in promoter regions, 13% and 6% in exonic regions and 4% and 3% in intronic regions, respectively (Suppl. Fig. S5). Using a Venn diagram, we identified 661 CpGs hypermethylation sites shared between all five sampling time points for CPK but no time specific CpG sites for all the three cultivars (Fig. 3C and Suppl. Fig. S6). While our study focused primarily on CpG methylation, CHG and CHH methylation were also detected but at lower levels (Fig. 3D). It is also noteworthy that if we compare the first three sampling time points (T = 0, T = 5 and T = 15) (Fig. 3C), differential methylation does not show a sharp increase between T = 0 and T = 5 as observed with SNPs. Instead, we observe a significant decrease in the number of methylated CpG sites, suggesting that the initiation of culture may have a lesser immediate impact on DNA methylation. However, it is possible to see slight increases and decreases in differential methylation levels over time (Fig. 3D).

### Gene ontology and pathway enrichment analyses

**Fig. 4:**
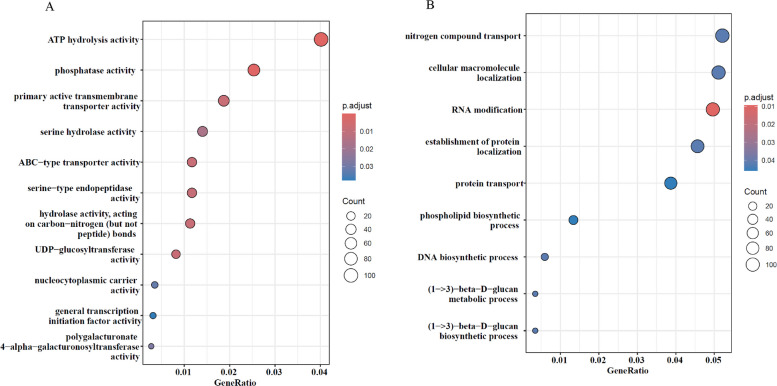
Gene ontology (GO) analysis of genes affected by genomic mutations in Critical Purple Kush (CPK) cultivar. **A** GO terms related to molecular function; **B** GO terms related to biological process

Gene ontology (GO) and KEGG pathway enrichment analyses were performed on the gene sets harboring the identified SNPs and DMPs. The most represented GO terms spanned the three categories: biological process (BP), cellular component (CC) and molecular function (MF) for all the genes from the three cultivars (Fig. 4). We examined enriched terms to identify those relevant to cannabis micropropagation. For genes harboring significant SNPs, 20 GO terms were found to be significantly enriched after false discovery rate (FDR) correction (*q* < 0.05), primarily in the molecular function and biological process categories. In the MF category, the most enriched terms include ATP hydrolysis activity, phosphatase activity, and ABC-type transporter activity (Fig. 4A), while in the BP category, enriched terms include RNA modification and nitrogen compound transport (Fig. 4B). Although no GO terms in the CC category remained significant after FDR correction, nine terms showed nominal significance (*P* < 0.05), mostly associated with organelle membranes and envelopes such as the bounding membrane of organelles, organelle envelope, and outer membrane (Suppl. Fig. 7A).

No pathways reached significance after FDR correction (*q* < 0.05) for the KEGG pathway analysis, however, 10 pathways showed unadjusted significance (*P* < 0.05), including those involved in metabolic pathways such as terpenoid backbone biosynthesis, fatty acid biosynthesis and folate biosynthesis, as well as cellular processing pathways like nucleocytoplasmic transport, protein export and base excision repair (Suppl. Fig. 7B).

Similarly, for genes associated with DMPs, functional enrichment analysis revealed 25 GO terms (8 in MF, 13 in BP, and 4 in CC) significantly enriched with unadjusted significance at* P* < 0.05 (Suppl. Fig. 8 A-C). Specifically, enriched MF terms included helicase activity, carbohydrate binding, and catalytic activity, acting on DNA, while enriched BP terms were related to monosaccharide metabolic process, regulation of flower development, and peptidyl-lysine modification. The CC category featured terms such as endosome, external encapsulating structure, proteasome regulatory particle, and clathrin coat. Additionally, five KEGG pathways were also enriched in this gene set (unadjusted significance at *P* < 0.05), primarily related to glutathione metabolism, alpha − linolenic acid metabolism, and taurine and hypotaurine metabolism (Suppl. Fig. 9).

### Distribution of somatic mutations and methylation patterns throughout the genome

**Fig. 5:**
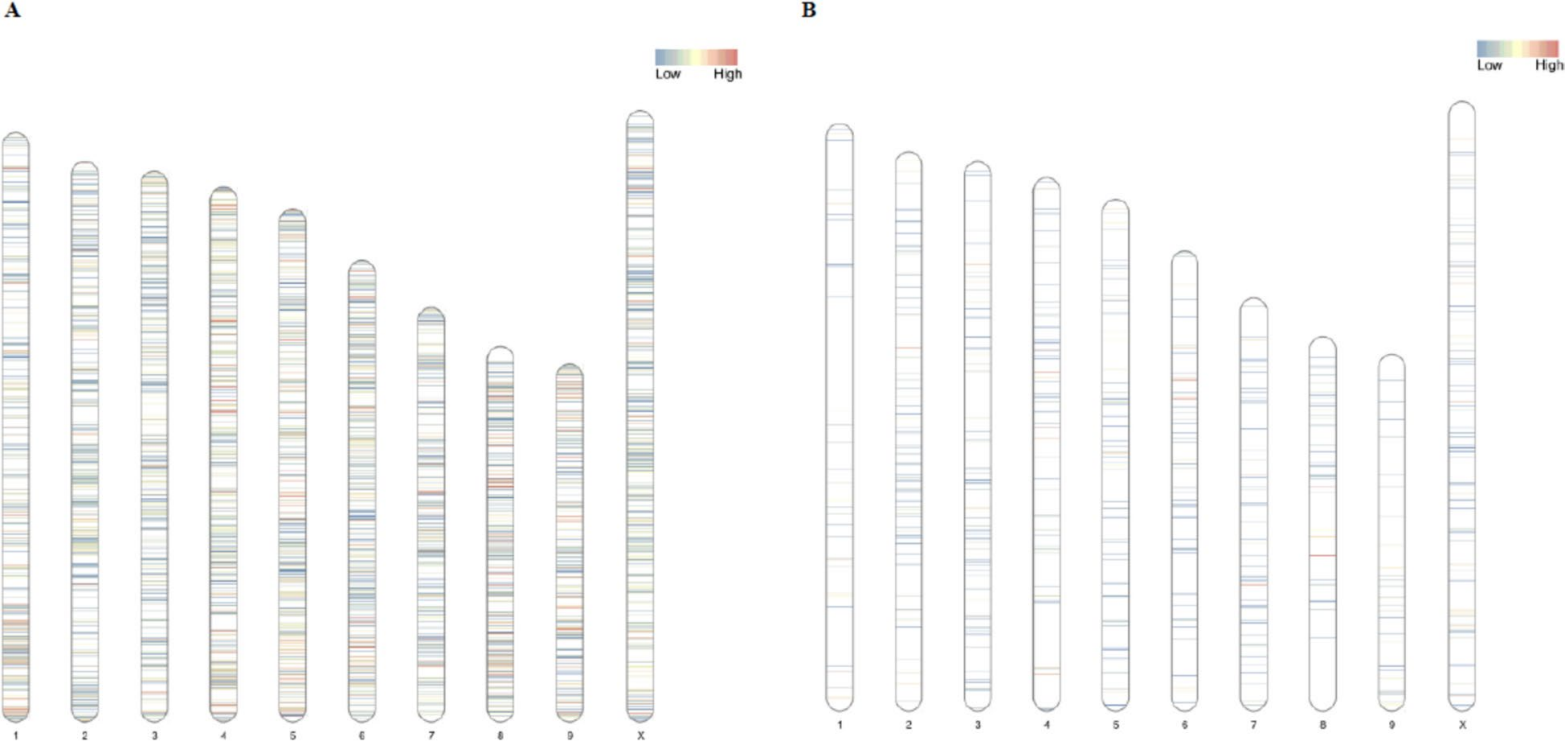
Distribution of genomic variants and DNA methylation sites across the cannabis genome of Critical Purple Kush (CPK) cultivar. **A** Single nucleotide polymorphisms (SNPs); **B** Differentially methylated positions (DMPs)

To verify if there were preferential sites for genetic variation or methylation, we plotted the chromosomal distribution of lowly and highly mutated genomic regions, as well as differentially methylated regions (Fig. 5A, B). This visual representation of the genetic and epigenetic modifications taking place in CPK samples throughout the experiment reveals that regions with low mutation density are the most common across the genome, although regions with high mutation density are quite frequent in chromosomes 1, 4, 5, 6, 8 and 9 (Fig. 5A). Comparatively, low and high differential CpG methylation levels are much less common than mutations, with most of the highly methylated regions residing in chromosome 4, 6, 7 and 8 (Fig. 5B).

## Discussion

The majority of studies on the genetic stability of cannabis in the context of micropropagation have not conducted long-term trials following multiple subcultures (Monthony et al. 2021), a gap we aimed to address in this study. Indeed, industrial scale micropropagation by legal producers is likely to imply long multiplication cycles, which we emulated by maintaining multiple cannabis cultivars in the vegetative phase for 60 weeks with 3-weeks-subclutures, allowing plantlets to grow multiple new shoots without outgrowing their vessels. Additionally, most studies assessing the genetic stability of in vitro cannabis have relied on multilocus marker-based genetic sequencing methods, which only detect mutations at specific sites and are therefore less representative of the genetic modifications throughout the whole genome (Lata et al. 2011; Ioannidis et al. 2022; Borin et al. 2025; Cull and Joly 2025). As a result, the true extent of in vitro mutation accumulation in cannabis remains unclear, despite its potential for significant impact on the desired phenotypes of regenerated plants. Therefore, we proposed a different experimental approach taking advantage of the genotyping by sequencing (GBS) technology, more specifically the 3D-GBS method to assess genome wide mutation accumulation since it allows an evenly distributed coverage of the genome compared to other marker-based methods while retaining the ultra-high-throughput genome-wide genotyping capabilities at a more affordable cost than conventional GBS or whole genome sequencing (de Ronne et al. 2023; Aina et al. 2025). Additionally, implementation of methylome sequencing via EM-seq method provided for the first time in the context of cannabis micropropagation, insights into DMPs and their long-term modifications. Among other things, we quantified mutation levels at various stages of micropropagation. Our results show that the mother plants initially had 12,943 (CPK), 12,460 (GC), and 13,145 (GEL) SNPs (Fig. 1). It is also noteworthy to mention that the sequencing reads were mapped to the CS10 v2.0 cannabis reference genome, which is inherently different from our studied cultivars and is thus responsible for part of the initial SNP counts (Grassa et al. 2021). Most interestingly, we observed a rise in mutation count between culture initiation (T = 0) and the 5th transplant (T = 5), with CPK, GC and GEL accumulating 1,587, 1,284 and 1,743 SNPs, respectively, over a four-month period. This mutation rate was unmatched throughout the duration of this experiment and was remarkably uniform across all three genotypes. In fact, only a slight increase in mutations were observed from the 5th (T = 5) to the 15th (T = 15) transplant, accumulating only 127, 176 and 111 additional mutations for CPK, GC and GEL, respectively, probably indicating that in vitro plantlets have acclimated to their environment by that point. This result is particularly important for growers considering micropropagation for industrial purposes. Indeed, our findings suggest that the 5th transplant mark could be regarded as the optimal period to assess various parameters such as plant regeneration, vigor, morphology and yield as part of a phenotyping program. First, this would allow growers to eliminate plantlets harboring deleterious mutations and could even lead to the development of new elite genotypes harboring advantageous mutations or, at least, neutral ones (non-deleterious). Second, our results indicate that the vegetative state could probably be maintained in vitro for at least 15 additional cycles (approximately one year) with minor risk to genetic stability and ultimately to flower phenotype reproducibility. Additionally, between the 15th (T = 15) and the 20th (T = 20) subculture cycles, we observed a slight decrease in the number of mutations in two out of three cultivars with CPK and GEL losing 15 and 158 SNPs, respectively, while GC barely gained 7 additional mutations. These very minor changes are likely a result of our bulk sampling method; however, they suggest that the genetic stability of in vitro plantlets can be maintained well over a year. Obviously, further research is needed to confirm these results over a longer period, but overall, our data suggest that micropropagation is a well-suited method for long-term industrial cannabis production.

In this study, we identified a substantial number of genetic variations sites during 60 weeks of in vitro cultivation. These variations were similarly distributed across the three cultivars: CPK (16,169), GC (15,472) and Gel (16,605), (Suppl. Table S1). In a similar study (Adamek et al. 2022) identified 592,000 unique variants in the top leaves of a 1.5-year-old mother plant of the cannabis cultivar Honey Banana using whole-genome sequencing (WGS). However, our results are not directly comparable to the former study since the WGS approach is expected to reveal more SNPs than GBS. In addition, their experimental design does not account for the temporal factor in the same way as ours, since their identified mutations are relative to the reference genome at a single time point, whereas we also considered de novo mutations that emerged throughout the multiple in vitro subcultures (Fig. 1).

Interestingly, our results seem to some extent contradict the genetic mosaicism hypothesis brought forward by Adamek et al. (2022), as we observed a sudden increase in mutations followed by a stationary phase from the 5th subculture onwards, suggesting that in vitro propagated plants, once they have acclimated to their new conditions, are more genetically stable than those obtained through cuttings. Otherwise, extrapolation of our results could provide an estimated number of mutations across the whole genome, which would most likely be around the same order of magnitude. Indeed, the expected fraction of the genome covered is roughly 1.2% with 3D-GBS (de Ronne et al. 2023). We measured the mean nucleotide diversity (π) of our 60-week-old samples to be around 2 × 10^–4^ for all cultivars (Suppl. Table S2). This value is of the same order of magnitude as that reported by Adamek et al. (2022), who found a mean nucleotide diversity of 6 × 10^–4^. The linkage disequilibrium (LD) decay curve shows a rapid decline of *r*^2^ within the first few kilobases (Suppl. Fig. S10). A similar pattern of rapid LD decay was also reported by Adamek et al. 2022), whereas extremely fast LD decay as been reported in wild cannabis populations by Aina et al. (2025).

To better understand the potential biological impact of these variants, the GO enrichment terms of SNP-associated genes were determined and show a partial overlap with those found in the DMP-associated genes, pointing to the processes being most likely influenced by both somatic and epigenetic mutations, especially in molecular function categories involving ATP-driven catalytic processes, lyase activity, ion and phospholipid binding and active transmembrane transport, highlighting a potential convergence in regulatory and metabolic pathways potentially shaped by both genetic variation and epigenetic modifications in the context of cannabis micropropagation.

Although we report significant over-representation for these terms, we must keep in mind that only a minority of the identified mutations are classified as high impact (0.1%). Therefore, the GO results serve more as an approximation of genetic targets that are more susceptible to being affected by nucleotide variations than other regions. On that note, the generation of precursor metabolites is probably one of the most important terms to consider in future studies due to its significant role in cannabinoid biosynthesis. Other terms mentioned above are not to be taken lightly either, as the biological machinery allowing cannabis plants to thrive and produce high-value compounds is highly complex and involves a plethora of underlying mechanisms. Our results highlight the importance of monitoring both genetic and epigenetic stability for long-term cannabis cultivation.

The principal component analysis (PCA) plots highlight temporal shifts in mutational patterns across cultivars, with certain time points (e.g., T5 or T10 depending on the cultivar) showing distinct profiles that may reflect biological or culture-induced mutation spikes. In contrast, CpG methylation patterns were more stable throughout this study. The PCA of the methylation data revealed a lack of clustering between CPK samples, although T = 0 and T = 5 are grouped together, as well as T = 15 and T = 20, suggesting a slower but steady change in methylation patterns over time (Fig. 3A). Molecular functions of DNA methylation in particular regions are still not clearly understood. For example, promoter DNA methylation, which is usually linked with gene transcription inhibition, can in some cases promote gene transcription instead (Zang et al. 2018a). We identified 79 shared CpG DMPs between all sampling time points for CPK, reinforcing the idea that DNA methylation changes may occur less frequently than mutations (Fig. 3C). However, methylation changes may be involved in regulating the expression of genes affected by mutations to minimize their impact on the plant’s overall health in the case of deleterious mutations. Furthermore, the shared CpG DMPs between the different time points of the same cultivar identified in this study support the results of other studies suggesting that epigenetic changes, such as DNA methylation, can persist across multiple generations, reinforcing the heritable nature of these modifications (Johannes et al. 2009; Cortijo et al. 2014; Virgen et al. 2025). A recent paper on methylation in micro-propagated cannabis using the novel comparative restriction enzyme analysis of methylation (CREAM) method laid some foundational work regarding specific methylation patterns (Boissinot et al. 2023). Despite the different sequencing methods used, one of the key limitations they encountered was the lack of different sampling time points, which they identified as a crucial component for future research, especially regarding the initiation of culture as plants are transitioning from regular growth conditions to in vitro conditions. Since we observed a rapid plateau in genetic mutations after the fifth generation, but a sustained accumulation of DMPs over time, we suggest that the decline in genetic stability of cannabis during micropropagation may be associated primarily with alterations in DNA methylation patterns. Our study provides valuable insight and complementary data on this subject, as we found that differential methylation levels do not spike between T = 0 and T = 5 as observed with SNPs. This suggests that the initiation of culture may have less impact on DNA methylation than previously thought. Instead, we noticed slight increases and decreases in differential methylation levels over time for all three cultivars studied. Thus, DNA methylation does not accumulate in a strictly progressive manner but instead appears to vary over time, potentially reflecting adaptative responses to growth conditions and, to some extent, could possibly be linked to the observed increase in mutational variants. Indeed, DNA methylation can be altered at individual loci or across the entire genome under environmental stress conditions (Zhang et al. 2018b). Although some CHG and CHH methylation were detected, we focused our attention on CpG methylation since the EM-seq method is better at detecting this type of modification (Vaisvila et al. 2021). Moreover, a notable finding result from our dataset is the difference between cultivars at the level of CpG methylation. For instance, we observed that CPK, GC and GEL respectively had 22%, 9% and 13% for promoter regions, 13%, 5% and 6% for exon regions, 4%, 4% and 3% for intron regions and 61%, 82% and 78% for intergenic regions, indicating a strong cultivar dependency in the region of accumulation of these types of epimutations.

Overall, DNA methylation in cannabis micropropagation is still new from a scientific standpoint, and further research should absolutely take it into account, especially in relation to mutational variants. Our results also demonstrate that both mutations and methylation changes seem to appear in specific genomic hotspots, with certain regions being clearly more prone to mutation accumulation while others remain largely unaffected. In this study, we were able to detect mutational variability during micropropagation, confirming our initial hypothesis that 3D-GBS is a cost-effective and reliable tool for monitoring genetic stability in in vitro cannabis culture. While our findings provide concrete, interpretable data on mutation rates during long-term cannabis micropropagation, we also uncovered evidence indicating that mutations do not accumulate indefinitely as cultivation progresses, instead they appear to plateau after five transplanting cycles, which is a highly valuable insight for growers investing in micropropagation systems.

In line with our second hypothesis, EM-seq proved to be an effective tool for methylation monitoring, which revealed that overall DNA methylation levels remain relatively steady throughout the different stages of micropropagation, although their nature seems to vary over time. Nevertheless, our results show that both mutation and methylation profiles differ across cultivars, implying that cultivar-dependent optimization and monitoring strategies are essential for the successful implementation of an industrial-scale micropropagation production systems.

## Conclusion

This study puts forth a new standard in the field of cannabis research, particularly in terms of sequencing methods and mutation rate analysis that are commonly used, as well as good propagation practices by in vitro culture. Indeed, knowing the mutation rate for each cultivar as well as its evolution over time can allow producers to better monitor their cultivars, allowing them to set up a storage system for genotypes with a desired phenotype (via cryopreservation, storage or other) and thus have the possibility to start over with the original genetic material at predetermined intervals to never lose the desired properties of a cultivar, therefore ensuring the preservation of the sought-after genetics over long periods of time. Naturally, there are still many unanswered questions, highlighting the pressing need for research surrounding the in vitro cultivation of cannabis as well as the preservation of valuable genotypes over even more extended periods. Among other things, it would be relevant to know more about the correlation between the specific genomic regions and the metabolic processes they regulate. In this way, better advice could be provided on the renewal of cultures from tissues containing the original genetic material. In addition, it would be essential to study cannabis plants that are already showing signs of performance decline to determine which genes are affected as well as the impact of somaclonal variations and DNA methylation on them. A combination of genomics, methylomics, proteomics, morphological trait monitoring and chemical analysis of the final products should provide a strong foundation for advancing future research surrounding industrial cannabis micropropagation.

## Materials and methods

### Plant materials and culture conditions

To establish and maintain in vitro cultures of three cannabis cultivars for long-term micropropagation and subsequent genetic and epigenetic analysis, high-THC cannabis mother plants of the cultivars Critical Purple Kush (CPK), Gelato (GEL) and Green Crack (GC) were grown in a greenhouse at Phytoclone *Inc*.'s facility (Saint-Étienne-des-Grès, QC, Canada) for 3.5 years before the experiment. These plants were originally sourced from ONO Cannabis Inc., located in Lévis, QC, Canada. For each mother plant, stems of approximately 4 cm with at least one bud were collected for disinfection and in vitro culture initiation. Explants were first thoroughly washed under running tap water for 5 min. Surface disinfection was performed with 2 drops of Tween-20 under constant agitation. They were then immersed in 70% (v/v) ethanol for a brief period and gently agitated. Surface disinfection was further performed in a separate container using 10% (w/v) sodium hypochlorite solution. After disinfection, explants were rinsed three times with sterile distilled water for 5 min each to remove residual disinfectant. Finally, necrotic or damaged extremities were carefully removed before transferring the explants to culture media under aseptic conditions. Once disinfected, the stems were placed on Acer TDZ initiation medium, which is adapted from (Kerns and Meyer 1986) and consists of Murashige Skoog (MS) medium supplemented with 0.1 mg/L thidiazuron at pH 5.8, then they were incubated for 1 week with lights off in a growth room maintained at 25 °C with 70% relative humidity and cool white lights (400 lumens, 12 h/12 h photoperiod). The plantlets were then transplanted onto Can TC propagation medium, which is adapted from (Lata et al. 2016) and (Damiano et al. 2005) supplemented with 0.5 mg/L gibberellic acid (GA3), 0.01 mg/L indole-3-butyric acid (IBA) and 0.002 mg/L Meta-topolin at pH = 5.7. These plantlets were then maintained on Can TC with transplanting intervals of 3 weeks for 60 weeks, with occasional passes on the MS IBA charcoal preservation medium adapted from (Richez-Dumanois et al. 1986) comprising of an MS base supplemented with 0.6 mg/L IBA and 500 mg/L activated charcoal at pH = 6.8. A summary table of all media used A summary table of all media used, including their composition, is provided in Supplementary Table 3. The 100 mg leaf samples were taken in duplicate for each cultivar at the time of culture initiation and after every 5th transplant, then frozen at −20 °C for later DNA extraction.

### DNA extraction

In this study, pooled samples were used to estimate the accumulation of genetic mutations and epigenetic changes (epi-mutations) across successive micropropagation generations in *Cannabis sativa*. For each cultivar (CPK, GC, and Gel) and each sampling stage, leaf tissue from 4–6 clonally propagated individuals was collected and pooled prior to genomic DNA extraction, with pools maintained separately for each genotype and time point. This sampling strategy enabled the estimation of allele frequencies and DNA methylation levels within each genotype, thereby assessing overall genetic and epigenetic stability across clonal individuals rather than inter-individual variability. Pooled plant tissue samples have been widely used in plant studies to quantify average DNA methylation and epimutation levels. Previous studies have shown that pooling multiple individuals provides accurate genome-wide allele frequency estimates when sequencing depth is sufficient, yielding results comparable to individual genotyping (Fracassetti et al. 2015; Rellstab et al. 2013).

 For genomic DNA extraction, frozen leaf tissues were ground using a Tissue Lyser (Qiagen, Tissue Lyser II) for two cycles of 1 min at 30 Hz in tubes containing a tungsten bead from the DNeasy plant mini kit, according to the manufacturer's instructions. The quantity and quality of the genomic DNA extracted were assessed using a Nanodrop spectrophotometer (Implen, Westlake Village, CA, USA) and DNA concentration was quantified with the Quant-iT Picogreen kit, which allows microplate assaying using a spectrofluorometer. The extracted DNA was stored at −20 °C until it was used for further analysis.

### Genotyping by sequencing (GBS)

To identify single nucleotide polymorphisms (SNPs) across the genomes of three cannabis cultivars using 3D-GBS technology, the extracted DNA samples were sent to the Genomic Analysis Platform (http://www.ibis.ulaval.ca/en/services-2/genomic-analysis-platform/) at the Institut de Biologie Intégrative et des Systèmes (IBIS) of Université Laval (Quebec, Canada), where Illumina libraries were prepared for each cultivar using the Illumina Tru-seq DNA Library Prep Kit, following the manufacturer's protocol. The quality of the generated libraries was verified on an Agilent Bioanalyzer 2100 system with a high-sensitivity microarray. The triple-digest 3D-GBS sequencing, making use of three restriction enzymes (PstI/NsiI/MspI), and EM-seq were performed on the Illumina Novaseq 6000 platform Genome Québec Innovation Center in Montreal, QC, Canada.

### Enzymatic methyl-sequencing (EM-seq)

To analyze DNA methylation patterns in the cannabis genome during micropropagation using EM-seq, DNA samples were sent to Genome Québec Innovation Center in Montreal, QC, Canada, for library preparation and sequencing. The paired-end EM-seq libraries were then sequenced on the Illumina Novaseq 6000 platform.

### 3D-GBS data analysis

To assess genetic diversity, mutation rates, linkage disequilibrium and functional enrichment of identified SNPs and DMPs were determined. The quality of the raw 3D-GBS reads was checked and verified using the FastQC software (Andrews 2023). They were then passed through the Fast-GBS bioinformatics pipeline (Torkamaneh et al. 2017) using the most complete and recognized reference genome available, cs10 v2.0 (GenBank Accession No. GCA_900626175.2) (Grassa et al. 2021). The Variant Call Format (VCF) files generated by FAST-GBS were filtered using TASSEL (Bradbury et al. 2007). The datasets were then handled using the R (R Core Team 2023) package gdsfmt (Zheng et al. 2012). Variants annotation was carried out using the SnpEFF software (Cingolani et al. 2012).

### Nucleotide diversity, mutation rates and linkage disequilibrium analyses

Nucleotide diversity (π) was calculated with the -window-pi option in VCFtools (Danecek et al. 2011), with a window of 10,000 bps with 1,000 bps step on the data set. A mean π value was used for all window calculations to obtain an average π value for the whole genome. The Linkage disequilibrium (LD) decay was determined using PopLDdecay software (Zhang et al. 2018a) based on the identified SNPs for each cultivar.

### Functional enrichment analysis

To further determine the biological relevance of the genes with identified SNPs and DMPs, Gene ontology (GO) analysis was performed using DAVID (Database for Annotation, Visualization and Integrated Discovery) online functional annotation tool (Sherman et al. 2022). To categorize and reduce the matched terms, the Plant-GOslim list in the CateGOrizer tool (Hu et al. 2008) was used with the accumulative count classification method. Additionally, for a better understanding of GO term enrichment, analyses for Biological Processes (BP), Cellular Components (CC), and Molecular Functions (MF), as well as Kyoto Encyclopedia of Genes and Genomes (KEGG) pathway were performed using the R packages topGO (Alexa and Rahnenfuhrer 2024), AnnotationHub (Morgan and Shepherd 2024) and clusterProfiler (Wu et al. 2021). Enrichment results were annotated across the three GO domains using the following thresholds: *pvalueCutoff* = 0.05, *pAdjustMethod* = "fdr" (false discovery rate) as a multiple testing adjustment method, and *qvalueCutoff* = 0.05.

### EM-seq data analysis

The quality of the raw reads files were first checked using FastQC software (Andrews 2023) and trimmed with Trim Galore (v0.4.2) (Krueger et al. 2023) to remove adapters and low-quality reads. The trimmed reads were then analyzed by Bismark (Krueger 2011) with default parameters as recommended by the authors for EM-seq data and using the reference genome, cs10 v2.0 (GenBank Accession No. GCA_900626175.2). The identification of differentially methylated positions (DMPs) between samples was carried out using the MethylKit R package (Akalin et al. 2012), and their distribution across different genomic features (promoter, intron, exon, and intergenic) was visualized using the genomation R package (Akalin et al. 2015). Differential methylation regions across each chromosome was visualized using the RIdeogram R package (Hao et al. 2020). To illustrate the shared and unique DMPs among the 5-time points, Venn Diagrams were generated using the VennDiagram package in R (Chen and Boutros 2011). We performed a principal component analysis (PCA) using the R packages SNPRelate (Zheng et al. 2012) to analyze variation among the samples for DNA methylation and genotype data in relation to sampling time points and genetic background.

## Supplementary Information

Supplementary Material 1. Supplementary Fig. S1. Principal component analysis of the genomic mutations at different time points. (A) for Green Crack (GC); (B) for Gelato (GEL) cultivars.

Supplementary Material 2. Supplementary Fig. S2. Venn diagram showing the overlap and unique SNPs at different time points. (A) Green Crack; and (B) Gelato cultivars.

Supplementary Material 3. Supplementary Fig. S3. Impact, type and genomic location of SNPs detected by 3D-GBS. For Green Crack (A, B, C;) and Gelato (D, E, F) cultivars.

Supplementary Material 4. Supplementary Fig. S4. Principal component analysis of DNA methylation data across different time points. (A) for Green Crack (GC); (B) for Gelato (GEL) cultivars.

Supplementary Material 5. Supplementary Fig. S5. Genomic distribution of differentially methylated positions (DMPs). (A) Green Crack; (B) Gelato cultivars.

Supplementary Material 6. Supplementary Fig. S6. Venn diagram showing the overlap and unique differentially methylated positions (DMPs) at different time points. (A) Green Crack; and (B) Gelato cultivars.

Supplementary Material 7. Supplementary Fig. S7. Gene ontology (GO) and KEGG pathway analysis of functional groups affected by genomic mutation. (A) GO terms related to cellular component; (B) KEGG pathways.

Supplementary Material 8. Supplementary Fig. S8. Gene ontology (GO) analysis of genes affected by epigenetic mutation. (A) GO terms related to molecular functions; (B) GO terms related to biological processes; (C) GO terms related to cellular components.

Supplementary Material 9. Supplementary Fig. S9. KEGG-based functional analysis of genes affected by epigenetic mutations.

Supplementary Material 10. Supplementary Fig. S10. Linkage disequilibrium (LD) decay (r2) plot against the genetic distance (Kb) based on the genomic variants of the Critical Purple Kush (CPK) cultivar.

Supplementary Material 11. Supplementary Table S1. Results of the statistical analyses performed on the 3D-GBS sequencing dataset of micro-propagated plantlets .

Supplementary Material 12. Supplementary Table S2. Nucleotide diversity in different cannabis cultivars after 60 weeks of in vitro culture.

Supplementary Material 13. Supplementary Table S3. Composition of culture media used in this study.

## Data Availability

Sequence data supporting the findings of this study have been deposited at Sequence Read Archive under BioProject accession no PRJNA1321113.
