# Supplementary figures and images for: Micropropagation of Cannabis sativa: genetic and epigenetic stability assessment over multiple generations
Source: J Cannabis Res. 2026 Feb 19;8:43. doi: 10.1186/s42238-026-00406-y (PMC13020208; doi:10.1186/s42238-026-00406-y)

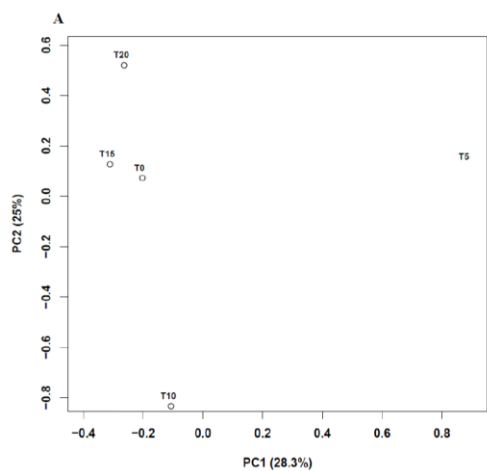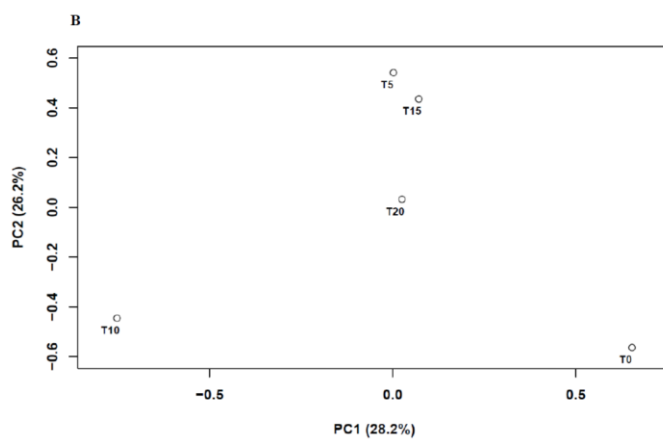

Supplement: Supplementary file 1 — Supplementary Material 1. Supplementary Fig. S1. Principal component analysis of the genomic mutations at different time points. (A) for Green Crack (GC); (B) for Gelato (GEL) cultivars. [file 42238_2026_406_MOESM1_ESM.pdf]

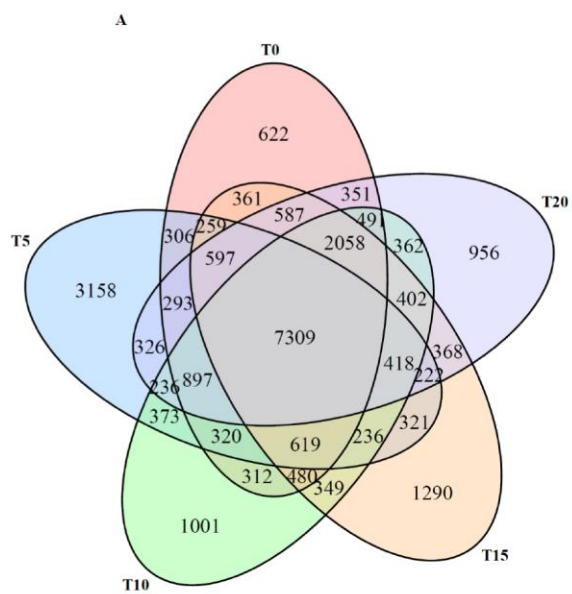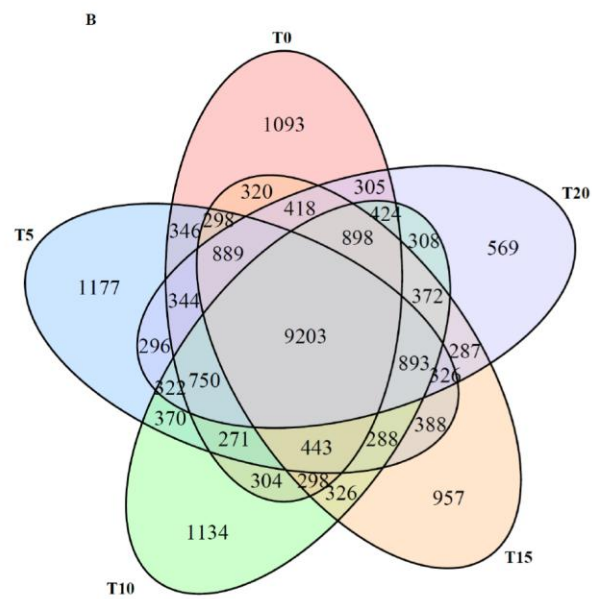

Supplement: Supplementary file 2 — Supplementary Material 2. Supplementary Fig. S2. Venn diagram showing the overlap and unique SNPs at different time points. (A) Green Crack; and (B) Gelato cultivars. [file 42238_2026_406_MOESM2_ESM.pdf]

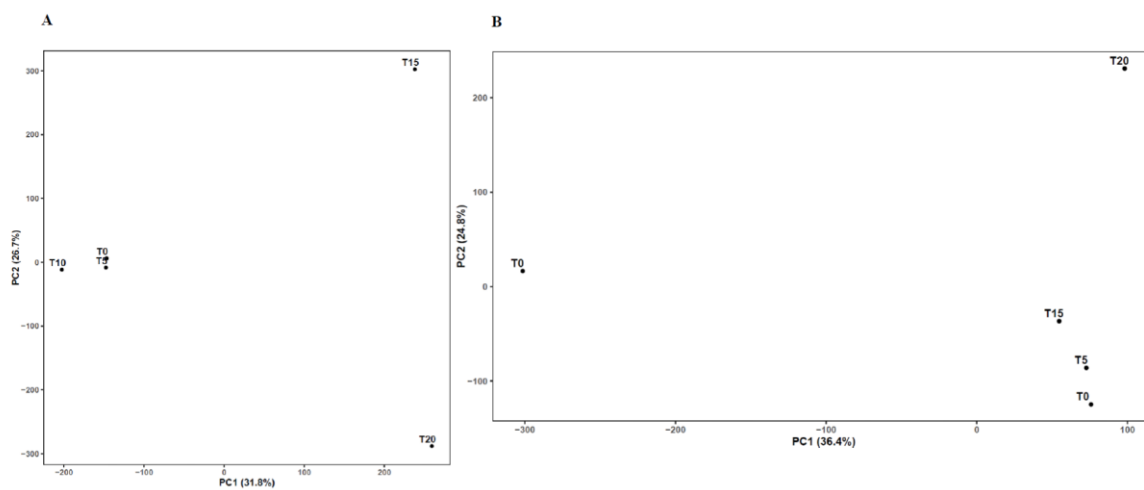

Supplement: Supplementary file 4 — Supplementary Material 4. Supplementary Fig. S4. Principal component analysis of DNA methylation data across different time points. (A) for Green Crack (GC); (B) for Gelato (GEL) cultivars. [file 42238_2026_406_MOESM4_ESM.pdf]

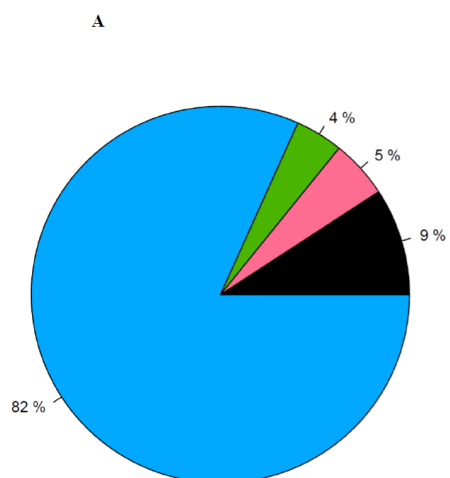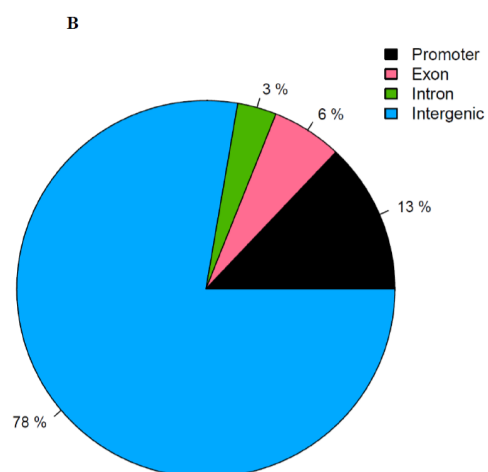

Supplement: Supplementary file 5 — Supplementary Material 5. Supplementary Fig. S5. Genomic distribution of differentially methylated positions (DMPs). (A) Green Crack; (B) Gelato cultivars. [file 42238_2026_406_MOESM5_ESM.pdf]

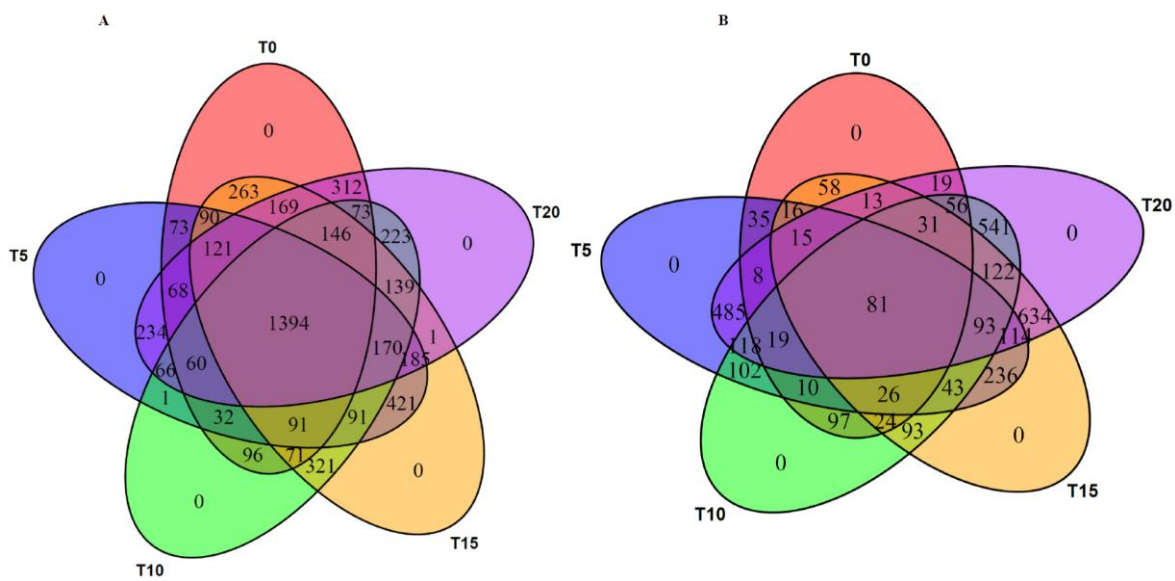

Supplement: Supplementary file 6 — Supplementary Material 6. Supplementary Fig. S6. Venn diagram showing the overlap and unique differentially methylated positions (DMPs) at different time points. (A) Green Crack; and (B) Gelato cultivars. [file 42238_2026_406_MOESM6_ESM.pdf]

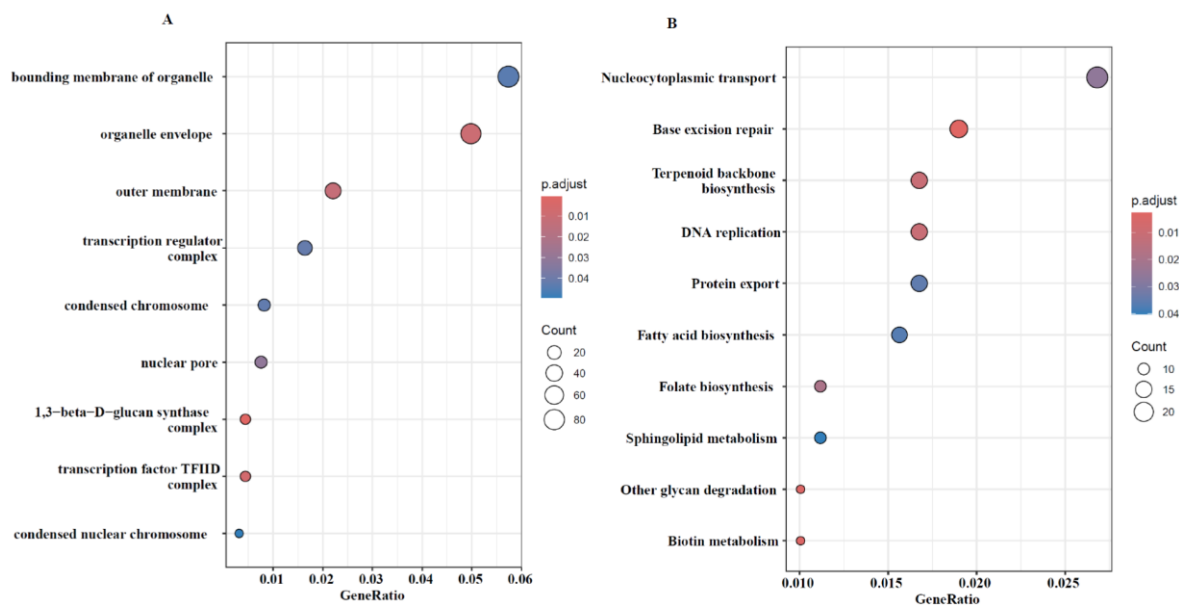

Supplement: Supplementary file 7 — Supplementary Material 7. Supplementary Fig. S7. Gene ontology (GO) and KEGG pathway analysis of functional groups affected by genomic mutation. (A) GO terms related to cellular component; (B) KEGG pathways. [file 42238_2026_406_MOESM7_ESM.pdf]

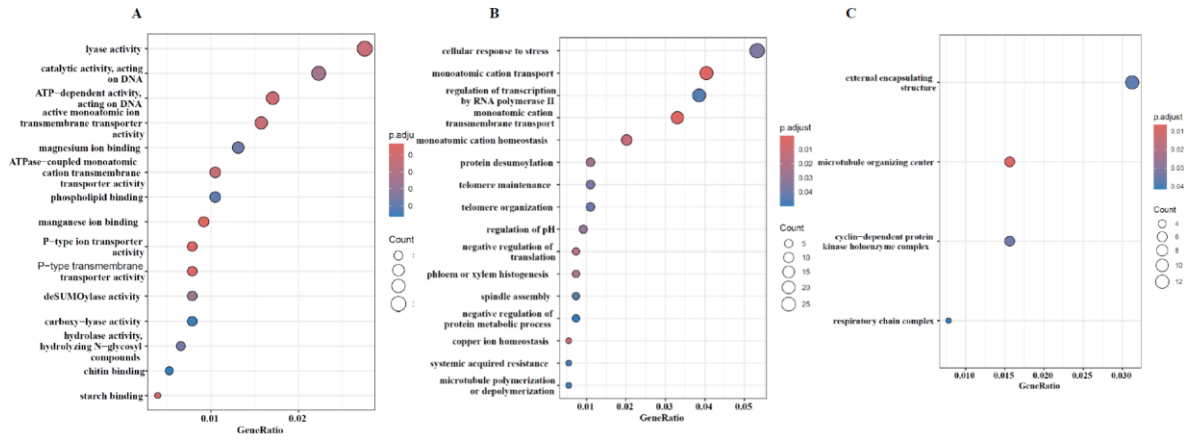

Supplement: Supplementary file 8 — Supplementary Material 8. Supplementary Fig. S8. Gene ontology (GO) analysis of genes affected by epigenetic mutation. (A) GO terms related to molecular functions; (B) GO terms related to biological processes; (C) GO terms related to cellular components. [file 42238_2026_406_MOESM8_ESM.pdf]

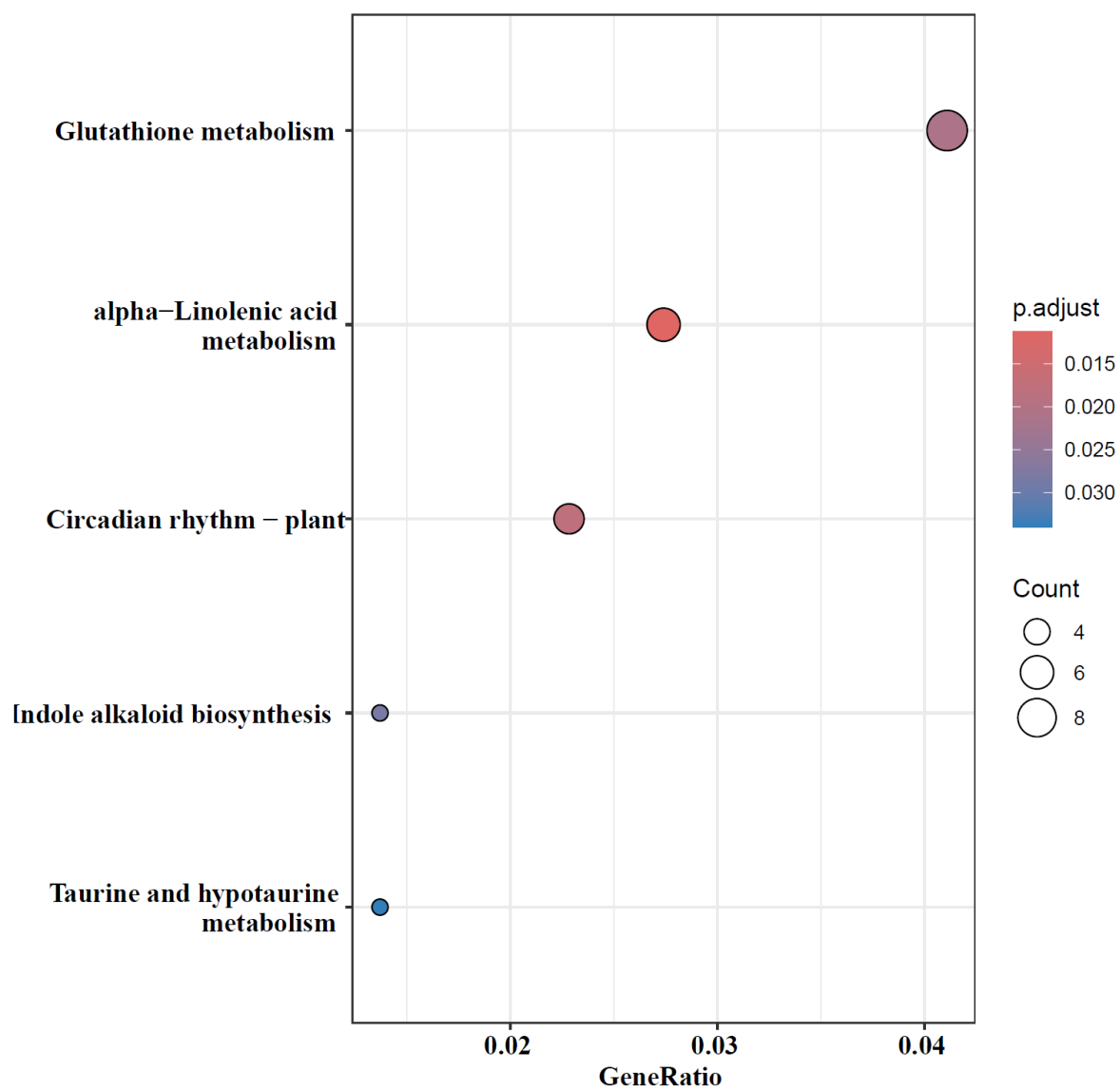

Supplement: Supplementary file 9 — Supplementary Material 9. Supplementary Fig. S9. KEGG-based functional analysis of genes affected by epigenetic mutations. [file 42238_2026_406_MOESM9_ESM.pdf]

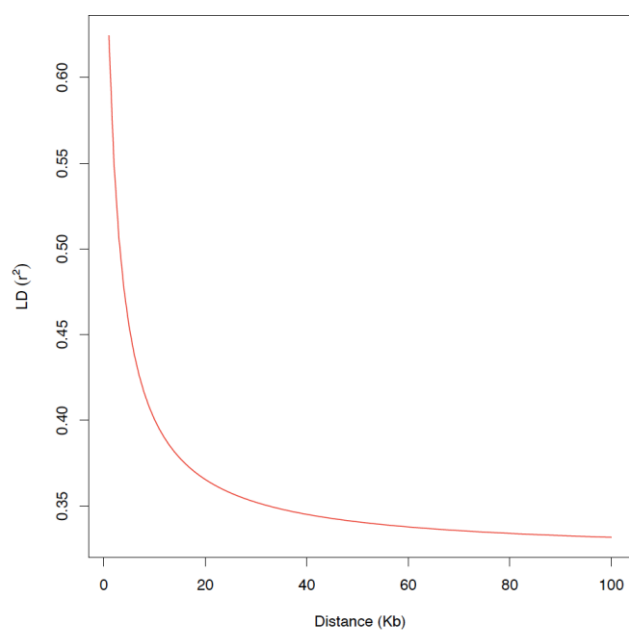

Supplement: Supplementary file 10 — Supplementary Material 10. Supplementary Fig. S10. Linkage disequilibrium (LD) decay (r2) plot against the genetic distance (Kb) based on the genomic variants of the Critical Purple Kush (CPK) cultivar. [file 42238_2026_406_MOESM10_ESM.pdf]
