# Supplementary material for: Micropropagation of Cannabis sativa: genetic and epigenetic stability assessment over multiple generations
Source: J Cannabis Res. 2026 Feb 19;8:43. doi: 10.1186/s42238-026-00406-y (PMC13020208; doi:10.1186/s42238-026-00406-y)
Supplement: Supplementary file 3 — Supplementary Material 3. Supplementary Fig. S3. Impact, type and genomic location of SNPs detected by 3D-GBS. For Green Crack (A, B, C;) and Gelato (D, E, F) cultivars. [file 42238_2026_406_MOESM3_ESM.pdf]

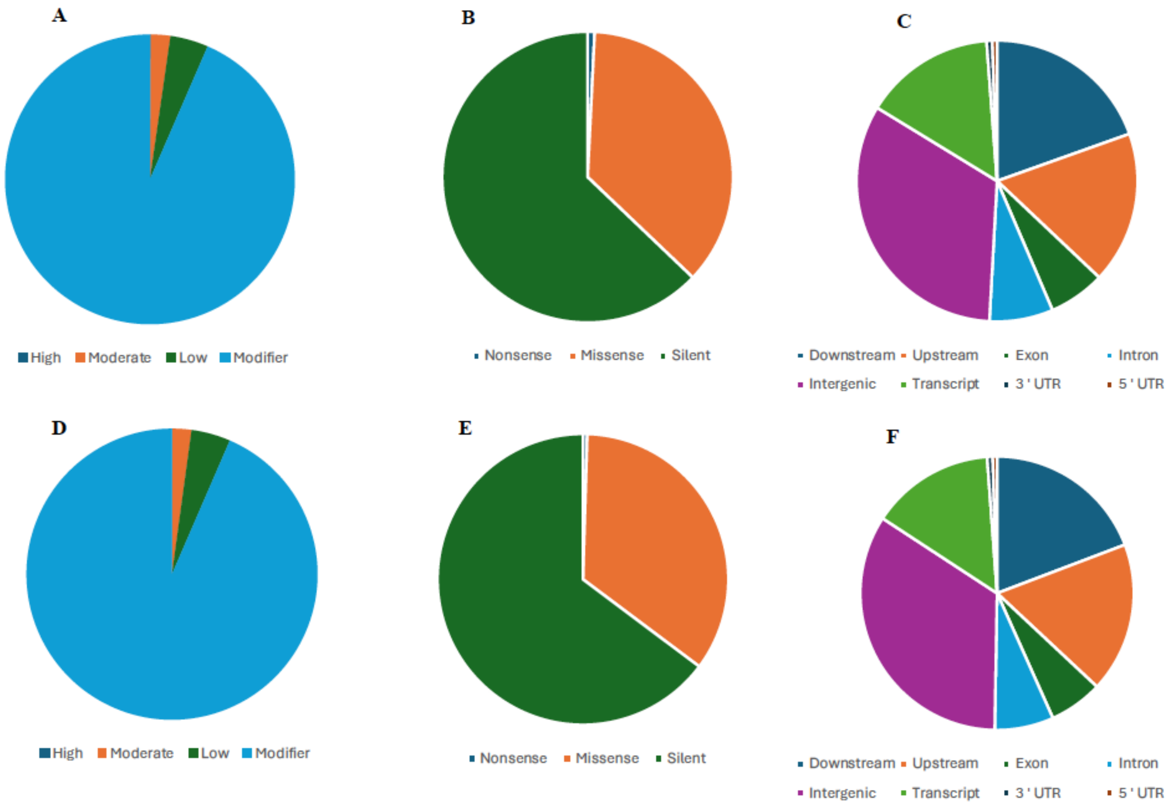
A
High
Moderate
Low
Modifier
B
Nonsense
Missense
Silent
C
Downstream
Upstream
Exon
Intron
Intergenic
Transcript
3 ' UTR
5 ' UTR
D
High
Moderate
Low
Modifier
E
Nonsense
Missense
Silent
F
Downstream
Upstream
Exon
Intron
Intergenic
Transcript
3 ' UTR
5 ' UTR
