# Supplementary material for: Micropropagation of Cannabis sativa: genetic and epigenetic stability assessment over multiple generations
Source: J Cannabis Res. 2026 Feb 19;8:43. doi: 10.1186/s42238-026-00406-y (PMC13020208; doi:10.1186/s42238-026-00406-y)
Supplement: Supplementary file 11 — Supplementary Material 11. Supplementary Table S1. Results of the statistical analyses performed on the 3D-GBS sequencing dataset of micro-propagated plantlets . [file 42238_2026_406_MOESM11_ESM.pdf]

Cultivar	Number of SNPs*	Variant rate	Missense/Silent ratio	Transition/Transversion ratio
Critical Purple Kush	16169	53640	0.5558	1.5503
Green Crack	15472	56032	0.5783	1.5568
Gelato	16605	52223	0.5367	1.5661

* Single nucleotide polymorphisms
