# Supplementary material for: Micropropagation of Cannabis sativa: genetic and epigenetic stability assessment over multiple generations
Source: J Cannabis Res. 2026 Feb 19;8:43. doi: 10.1186/s42238-026-00406-y (PMC13020208; doi:10.1186/s42238-026-00406-y)
Supplement: Supplementary file 12 — Supplementary Material 12. Supplementary Table S2. Nucleotide diversity in different cannabis cultivars after 60 weeks of in vitro culture. [file 42238_2026_406_MOESM12_ESM.pdf]

**Cultivar**	**Mean nucleotide diversity (π)**
Critical Purple Kush	2.0777×10^{-04}
Green Crack	2.0076×10^{-04}
Gelato	2.1739×10^{-04}
