# Supplementary material for: Micropropagation of Cannabis sativa: genetic and epigenetic stability assessment over multiple generations
Source: J Cannabis Res. 2026 Feb 19;8:43. doi: 10.1186/s42238-026-00406-y (PMC13020208; doi:10.1186/s42238-026-00406-y)
Supplement: Supplementary file 13 — Supplementary Material 13. Supplementary Table S3. Composition of culture media used in this study. [file 42238_2026_406_MOESM13_ESM.docx]

**Supplementary Table S3. Composition of culture media used in this study**

| **Media Components** | **Acer TDZ** | **Can TC** | **MS IBA Charcoal** |
| --- | --- | --- | --- |
| NH_4_NO_3_ (mg/L) | 16.5 | 1420 | 1650 |
| KNO_3_ (mg/L) | 19 | - | 1900 |
| MgSO_4_*7H_2_O (mg/L) | 3.7 | 740 | 370 |
| KH_2_PO_4_ (mg/L) | 1.7 | 260 | 170 |
| CaCL_2_*2H_2_O (mg/L) | 4.4 | 670 | 440 |
| Myo-Inositol (mg/L) | 1 | 100 | 100 |
| Riboflavin (mg/L) | - | 0.21 | - |
| Thiamine HCL (mg/L) | 0.1 | 0.6 | 0.1 |
| Nicotinic acid (mg/L) | 0.5 | 1.15 | 0.5 |
| Pyridoxine HCL (mg/L) | 0.5 | 0.6 | 0.5 |
| Ascorbic acid (mg/L) | - | 1 | - |
| α-Tocopherol (mg/L) | - | 0.0016 | - |
| H_3_BO_3_ (mg/L) | 6.2 | 4.8 | 6.2 |
| MnSO_4_*4H_2_O (mg/L) | 22.3 | 44.61 | 22.3 |
| ZnSO_4_*7H_2_O (mg/L) | 8.6 | 16.45 | 8.6 |
| Potassium Iodide (mg/L) | 0.83 | - | 0.83 |
| Na_2_MoO_4_*2H_2_O (mg/L) | 0.25 | 0.39 | 0.25 |
| CuSO_4_*5H_2_O (mg/L) | 0.03 | 0.25 | 0.03 |
| CoCl_2_*6H_2_O (mg/L) | 0.03 | - | 0.03 |
| Glycine (mg/L) | 2 | 0.86 | - |
| Na_2_EDTA (mg/L) | 37.25 | 37.25 | 37.25 |
| FeSO_4_*7H_2_O (mg/L) | 27.6 | 27.6 | 27.6 |
| Indole-3-butyric acid (mg/L) | - | 0.01 | 0.6 |
| Thidiazuron (mg/L) | 0.1 | - | - |
| Gibberellic Acid (mg/L) | - | 0.5 | - |
| Ca(NO_3_)_2_*4H_2_O (mg/L) | - | 1960 | - |
| K_2_SO_4_ (mg/L) | - | 1560 | - |
| Adenine (mg/L) | - | 20 | - |
| meta-Topolin (mg/L) | - | 0.002 | - |
| Activated Charcoal (mg/L) | - | - | 500 |
| Sucrose (g/L) | 30 | 20 | 30 |
| TC gel (g/L) | 4 | 4 | 4 |
| pH | 5.8 | 5.7 | 6.8 |
